# *Euphorbia hirta* L. as a potential resistance-modifying agent against ESKAPE pathogens: a systematic review

**DOI:** 10.3389/fphar.2026.1862731

**Published:** 2026-07-09

**Authors:** Kamran Zaman, Shivani Tendulkar, Kalesh Karun, Flemin Felix, Kranthi Kiran Akula, Nidhi Hiremath, Surthi Ravedar, Tejaswini Salunkhe, Jainabbi Patel, Asif Kavathekar, Jyothi Bhat

**Affiliations:** ICMR National Institute of Traditional Medicine (ICMR-NITM), Belagavi, Karnataka, India

**Keywords:** antimicrobial resistance, bioactive phytochemicals, resistance modulation, resistance-modifying agents, pharmacological activity, Euphorbia

## Abstract

**Background:**

The growing issue of antimicrobial resistance (AMR) in bacteria, particularly among the ESKAPE pathogens, has prompted the need to explore additional treatment options. *Euphorbia hirta* L. (*E. hirta*) is a botanical drug plant with antimicrobial activities attributed to its biologically active metabolites, namely flavonoids, terpenoids, and phenolic acids.

**Methods:**

This systematic review followed PRISMA guidelines to assess the antimicrobial effects of *E. hirta* against critical bacterial pathogens. A systematic search and collection of data from 27 peer‐ reviewed articles on the antimicrobial potential of *E. hirta* against different bacteria were conducted. Extraction solvents such as ethanol, methanol, petroleum ether, ethyl acetate, chloroform, and water were considered in this systematic review. The antimicrobial potential of *E. hirta* was assessed using methods such as MIC, MBC, and diffusion assays (disk and well diffusion).

**Results:**

Petroleum ether extract of *E. coli, S. aureus*, and *P. aeruginosa* showed the lowest MICs (0.4, 0.125, and 0.4 mg/mL, respectively), while ethanol extracts exhibited potent activity against S. aureus (14.53 mg/mL), K. pneumoniae (19.73 mg/mL), *P. aeruginosa* (20.06 mg/ mL), and *E. faecalis* (29 mg/mL). Chloroform extracts showed a relatively constant moderate level of activity against most of the organisms tested. It ranged from 1.45 mg/mL for *E. coli* and *P. aeruginosa*. In general, non-polar solvents such as petroleum ether and chloroform provided better results than their polar counterparts, like methanol (up to 100 mg/mL).

**Conclusion:**

The reported in vitro interactions between *E. hirta* fractions and conventional antibiotics, such as the observed reduction in MICs for cefepime and ciprofloxacin, suggest a potential resistance‐modifying effect. However, these findings remain preliminary and unsubstantiated for clinical translation. Future research should prioritize bioassay‐guided isolation, mechanistic studies and the development of standardized formulations for its integration into clinical practice for addressing antimicrobial resistance.

**Systematic Review:**

website, identifier CRD420251025371.

## 1 Introduction

Infectious diseases remain a primary cause of global mortality, a crisis now compounded by the escalating threat of antimicrobial resistance (AMR). Since the “golden age” of antibiotic discovery, the inhibitory potential of conventional antibiotics has been systematically compromised by the emergence of multidrug-resistant (MDR) pathogens (Singh and Kumar, 2013). AMR was estimated to cause 4.71 million deaths (95% UI 4.23–5.19) globally, including 1.14 million (1.00-1.28) directly attributable to AMR, with the highest numbers in sub-Saharan Africa and South Asia. AMR-attributable deaths declined by >50% in children <5 years of age but increased by >80% in adults aged ≥70 years between 1990 and 2021, driven by population ageing and rising carbapenem resistance in Gram-negative bacteria. Forecasts suggest 1.91 million (1.56-2.26) attributable AMR deaths are predicted to occur by 2050 (Naghavi et al., 2024). This shift in the paradigm has led to a critical evaluation of multi-target scaffolds and plant-derived secondary metabolites as the basis for the discovery of newer antibiotics (Angelini, 2024; Xu et al., 2026). Among the medicinal plants studied, the *Euphorbia* genus of the *Euphorbiaceae* family, the largest genus with 2,000 species, is recognised worldwide for its pharmacological potential (Mavundza et al., 2022).

**FIGURE 1:**
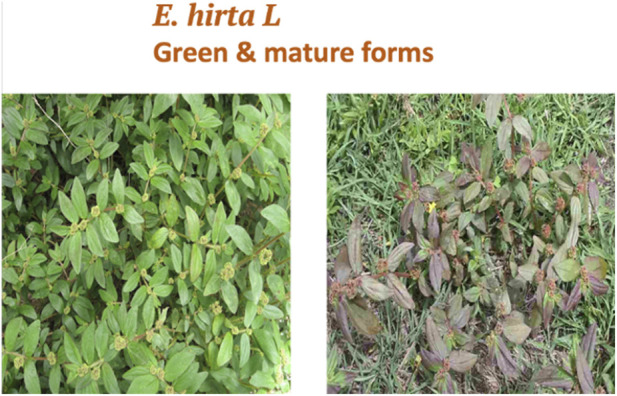
Green and mature forms of *Euphorbia hirta L*., illustrating variation in leaf pigmentation and growth stage.

*Euphorbia hirta L. (E.hirta)* is an annual herbaceous weed that belongs to the *Euphorbiaceae* family, also known as “Asthma plant”, “Dudhi” (Hindi), or “Ara tanah” (Malaysian). This botanical drug is native to Central America and has since naturalized across tropical and subtropical regions of Asia, Africa and the Pacific islands (Patil et al., 2009). The plant is semi-erect or prostrate, with a branch length of 60 cm, with a hairy stem ranging in colour from red to purplish. The leaves are characterized by serrated edges, simple, opposite, elliptic to oblong, and usually possess a purplish splotch close to the midrib (Asha et al., 2014; Srivastava et al., 2019). Its inflorescence is made up of a cyathium, an axillary or terminal cluster of unisexual flowers (Figure 1). The fruit of the plant is a hairy, three-lobed capsule with wrinkles, prismatic seeds, and blooms throughout the year (Liyongo Inkoto et al., 2020).

The ethnopharmacological history of *E. hirta* is extensive, with its white, milky latex and whole-plant decoctions being integral to Ayurvedic, Siddha, Unani, and traditional African medical systems. Traditionally, it has been mainly employed to treat respiratory complaints such as asthma, bronchitis, and cough, but is also widely used to treat various gastro-intestinal disorders (including amoebic dysentery and diarrhoea), skin disorders (warts, wounds, and boils) and genitourinary infections (gonorrhea) (Mavundza et al., 2022; Ghosh et al., 2025).

*E. hirta* has an extremely complex phytochemical composition, which contributes to its high levels of therapeutic potential. Important metabolite of *E. hirta* include terpenoids, polyphenols, flavonoids, antioxidants, and volatile oil molecules. It contains phenolic acids like gallic, caffeic, and chlorogenic acids; triterpenoids like α-/β-amyrin and taraxerol; flavonoids like kaempferol, quercetin, rutin, epicatechin 3-gallate, and myricitrin; and phytosterols like campesterol and stigmasterol. Hymenoxin, quercetin 3-O-arabinofuranoside, and n-butyl-L-rhamnopyranoside, are the valuable phytochemical metabolites (Wu et al., 2012; Asha et al. 2014). The plant has immunomodulatory, anti-inflammatory, and anticancer qualities in addition to its potent capacity to prevent the production of nitric oxide (NO), making it an efficient antioxidant. E. hirta has shown antibacterial activity against bacterial pathogens like *E. coli*, *S. aureus* and *P. aeruginosa*. Taking this into account, the botanical drug has potential for application in natural product-based medication development (Subali et al., 2024).

ESKAPE pathogens (*E. faecium, S. aureus, K. pneumoniae, A. baumannii, P. aeruginosa, and Enterobacter spp*.) pose a severe global health threat due to their capacity to establish multidrug-resistant (MDR) and pandrug-resistant (PDR) profiles through processes such as enzymatic degradation, porin loss, and biofilm formation. The World Health Organization (WHO) has designated these pathogens as “Priority 1: Critical” or “Priority 2: High” pathogens. These organisms are becoming more resistant to reserve antibiotics such as vancomycin and carbapenems and account for up to 70% of clinical cases in places like India (WHO, 2024).

“One Health” strategy is needed to address the spread of resistance in the human, animal, and environmental sectors in order to curtail the estimated 10 million annual deaths by 2050 (Naghavi et al., 2024). *E. hirta* has gained attention as a promising plant-derived candidate for addressing the global AMR crisis. Its bioactive secondary metabolites, especially phenolic acids and flavonoids, have antibacterial qualities that can break down bacterial membranes and overcome resistance. These activities may enhance the anti-microbial effects of conventional antibiotics against resistant pathogens. As a result, *E. hirta* merits additional research as an eco-friendly treatment approach to address AMR (Naghavi et al., 2024).

Although there are general ethnopharmacological accounts of *E. hirta*, this work offers the first quantitative and systematic synthesis that focuses on key pathogens. We assess the plant as a resistance-modifying adjuvant that can revitalise current antibiotics and support the One Health approach, rather than only as a stand-alone antimicrobial.

## 2 Methodology

### 2.1 Protocol registration

This systematic review was conducted in strict adherence to the PRISMA (Preferred Reporting Items for Systematic Reviews and Meta-Analyses) guidelines. The review protocol was prospectively registered with PROSPERO under the registration no. CRD420251025371.

### 2.2 Systematic search strategy

A comprehensive literature search was executed across four primary electronic databases: PubMed, Scopus and ScienceDirect. The following is a predefined search strategy outlined in Supplementary Table S1. In addition, Google Scholar was employed as an extra database to carry out the literature search. The literature search was carried out using the predefined strategy based on Boolean operators and keywords such as “*Euphorbia hirta*”, “Antimicrobial activity” and “ESKAPE pathogens”.

### 2.3 Article selection

A three-step selection method was followed in the selection of the studies for inclusion in the systematic review. The selection method involved the screening of the titles and abstracts of the studies. This was followed by the screening of the full text of the studies. The selection method was carried out independently in April 2025 and December 2025.

### 2.4 Inclusion criteria

A systematic review of the antimicrobial activity of *E. hirta* on ESKAPE pathogens will be carried out. This will be done through *in vitro* microbiological tests, where the botanical drug will be directly used as part of the experimental study.

### 2.5 Exclusion criteria

Studies were excluded based on a lack of information due to the pathogens not being classified as ESKAPE or a priority pathogen. The exclusion criteria included studies not written in English, case reports, case studies, *in vivo* and conference abstracts.

### 2.6 Data extraction & synthesis

Two reviewers screened all the titles and abstracts of the retrieved articles independently for potentially relevant studies. All the short-listed articles were carefully examined for eligibility. The reference lists of these articles were examined for any research that might have been missed in the initial search. The systematic review only considered peer-reviewed articles available in English for consistency in content. Two reviewers independently extracted trial characteristics for inclusion in this systematic review: last name of the first author, publication year, plant species used in the study, country of origin, type of solvent used, extraction method used, and type of antimicrobial assay and interpretation used (e.g., minimum inhibitory concentration (MIC), minimum bactericidal concentration (MBC)). The well diffusion method and disk diffusion method were also considered for inclusion in this systematic review. The data were analyzed, and all the values were compiled in the supplementary tables, which are referred to in the main manuscript. All the data was manually recorded using Microsoft Excel for consistency in documentation. In case of a discrepancy in results from both reviewers, a third reviewer was consulted for accuracy in the recorded information.

### 2.7 Assessment of risk-of-bias and reliability

The quality of the methodology and reliability of the data used in the papers under review were evaluated by the ToxR tool. This evaluation instrument includes 18 binary indicators classified into five categories, including (i) information on test substance; (ii) description of test system; (iii) description of study design; (iv) reporting of results; (v) plausibility of study design and results. Scoring was performed with one point for a positive answer and 0 points for a negative answer (Schneider et al., 2009; Kandhare et al., 2019).

### 2.8 Statistical analysis

The extracted data were organised using Microsoft Excel and analysed in IBM SPSS Statistics. Due to substantial methodological heterogeneity and insufficient data for quantitative synthesis, a formal meta-analysis was not performed. Instead, antimicrobial outcomes (MIC, MBC, DD, and WD) were summarised descriptively. For each microorganism–solvent combination, the number of observations, minimum, maximum, mean, and standard deviation of the reported study means were calculated. These statistics were used to describe the distribution of reported values across studies and do not represent pooled meta-analytic estimates.

## 3 Results

### 3.1 Selection of included studies

**FIGURE 2:**
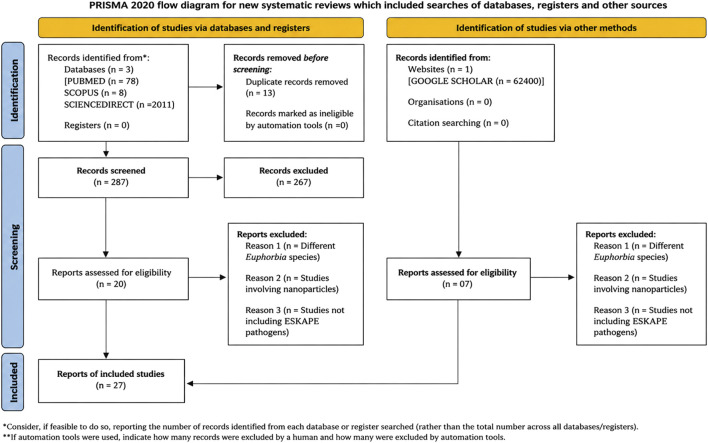
PRISMA workflow diagram depicting the study selection process for the systematic review.

A total of 287 articles were retrieved from a comprehensive search in PubMed, Scopus, and ScienceDirect databases [PubMed: 78; Scopus: 8; ScienceDirect: 201]. A total of 62,400 hits were obtained from Google Scholar search results, out of which the first 200 results were screened for eligibility based on relevance, resulting in 07 articles for this study. After excluding articles based on duplication, review articles, and non-*E. hirta* species, and non-ESKAPE focus, a total of 27 articles were found eligible for qualitative synthesis (Figure 2). The above PRISMA diagram shows the stringent selection criteria for this study, where only high-quality *in vitro* studies on *E. hirta* extracts against priority pathogens by standardized methods (MIC, MBC, DD, WD) were considered for inclusion in this review.

### 3.2 Study characteristics

The studies included in the review were from diverse geographical locations. However, the majority of the studies came from the Indian setting (12 studies, 44.4%), followed by other Asian nations (9 studies, 33.3%) like Malaysia, Pakistan, Philippines, Saudi Arabia, Vietnam, Yemen, and African nations (6 studies, 22.2%) like Nigeria, Tanzania, and the Democratic Republic of Congo. The year of publication of the studies varied from 1999 to 2025. It was observed that 77.8% of the studies (21 out of 27 dated studies) were published after 2010 (Supplementary Table S2).

Plant material utilization showed considerable variability, with leaves being the most investigated plant part (n = 12, 44.4%), followed by aerial parts (n = 8, 29.6%), and whole plant parts (n = 7, 25.9%). Maceration-based techniques, such as maceration and soaking, were the most investigated methods for plant material utilisation (n = 18, 66.7%), followed by Soxhlet extraction and specialised techniques such as percolation, accelerated solvent extraction, and chromatography. Various organic solvents, such as hexane, dichloromethane, chloroform, ethyl acetate, petroleum ether, methanol, ethanol, and acetone, along with water, were used in single or multiple combinations for extraction purposes. Among the solvents used, methanol, ethanol, and water were the most frequently used, mainly due to their polarity and ability to extract diverse bioactive secondary metabolites. Several studies have shown that successive solvent extraction achieves maximum recovery of phytochemicals from the plant material.

A stepwise method was used to evaluate the antimicrobial activity in each of the 27 analysed studies. MIC and MBC values against priority pathogens were determined using broth microdilution, although disk diffusion and well diffusion assays were mostly utilized for preliminary screening. A subset of studies extended beyond primary screening to include time-kill kinetics, checkerboard assays, and growth-inhibition percentage measurements to explore bactericidal dynamics and potential synergism between plant extracts and conventional antibiotics. FIC indices or equivalent combination metrics were used in these synergy-focused investigations to classify interactions as additive, synergistic, or indifferent. Sequential chromatography was carried out in a few studies to further purify the bioactive peptide fractions. The majority of the research utilised appropriate positive controls through the use of standard antibiotics. In many cases, the researchers have selected antibiotics based on the resistance profile of the test organisms. A few commonly used reference drugs included the use of broad-spectrum antibiotics such as ciprofloxacin, gentamicin, amoxicillin, ampicillin, streptomycin, and tetracycline. Additionally, the research included the use of cephalosporins such as cefepime, ceftriaxone, and ceftazidime. The research also included the use of carbapenems such as imipenem and meropenem. Furthermore, the research included the use of other relevant antibiotics such as vancomycin, chloramphenicol, and flucloxacillin.

The bacterial spectrum in the studies ranged from a variety of Gram-positive and Gram-negative bacteria, including the ESKAPE organisms. *E. coli* was the most commonly studied species, including the commonly occurring ATCC/MTCC strains and the more virulent uropathogenic and enteric pathogenic strains. *S. aureus*, including methicillin-resistant strains in some cases, *P. aeruginosa, K. pneumoniae, E. faecalis*, and *A. baumannii* were also commonly investigated in the studies, owing to the severe nature of the infections they cause in the urinary system, respiratory system, and in nosocomial infections. Some studies were particularly designed to test the pharmacological potential of *E. hirta* extracts on MDR phenotypes such as ESBL-positive *Enterobacteriaceae* and carbapenem-resistant non-fermenters in combination with other antibiotics.

According to the ToxR Tool assessment, 20 studies (74.1%) scored 15-18/18, five studies (18.5%) scored 11-14/18, and two studies (7.4%) scored <11/18. The median quality score was 16/18 (range: 10–17). The most frequently unmet criterion was reporting the purity of the test substance, while information regarding statistical analysis, control groups, replication procedures, and study design justification was also inconsistently reported. Most studies adequately described the test substance, test system, experimental procedures, and outcome measurements, supporting the overall reliability of the evidence base (Supplementary Table S3). While 74.1% of the studies achieved high numerical scores on the ToxRTool assessment, this metric primarily reflects the reporting of experimental parameters rather than the pharmacological validity of the results. Critically, none of the included studies performed specific assays to rule out pan-assay interference compounds (PAINS)-related artifacts or confirmed the stability of the isolated metabolites under assay conditions. Therefore, while the reporting is generally consistent, the evidence base for specific antimicrobial mechanisms remains weak.

### 3.3 Antimicrobial activity of *E. hirta*

The Minimum Inhibitory Concentration (MIC) is defined as the lowest concentration of *E. hirta* extract that prevents visible microbial growth, while the Minimum Bactericidal Concentration (MBC) is the lowest concentration that results in a 100% kill rate of the initial bacterial inoculum.

#### 3.3.1 Minimum inhibitory concentration (MIC)

**FIGURE 3:**
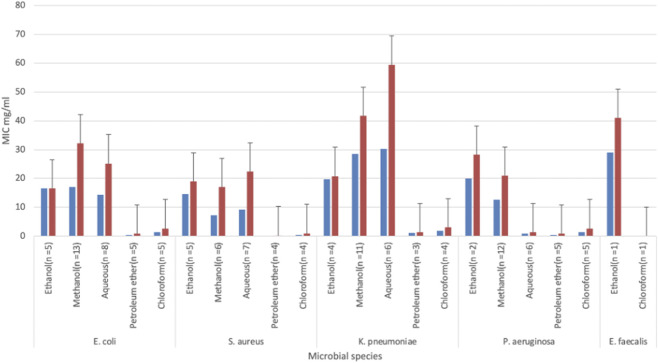
Minimum Inhibitory Concentration (MIC) of plant extract solvents against ESKAPE Pathogens.

MIC values ranged from a minimum (lowest potency, highest value) of 100 mg/mL (*E. coli* and *K. pneumoniae* with methanolic extracts) to a maximum potency (lowest value) of 0.125 mg/mL (*S. aureus* with petroleum ether extract of *E. hirta*). The highest SD was 41.0122 mg/mL for *E. faecalis* (ethanolic extract), indicating substantial variability. Ethanolic extracts demonstrated the most stable pharmacological potential and showed the narrowest range for *E. coli* (MIC 0.189–30 mg/mL, n = 5) compared to methanolic extracts (0.5–44 mg/mL, n = 13). Aqueous extracts showed a wider range (3–60 mg/mL, n = 8); while the *E. hirta* extracts using non-polar solvents like petroleum ether and chloroform consistently underperformed, often requiring concentrations exceeding 2 mg/mL to inhibit growth (with means of 0.40 ± 0.89 mg/mL (n = 5) and 1.45 ± 2.72 mg/mL (n = 5), respectively). Against *S. aureus*, methanolic extract was the most potent (mean 7.20 ± 17.04 mg/mL, n = 6) versus ethanolic extract (14.53 ± 19.02 mg/mL, n = 5), and for *K. pneumoniae*, methanolic extract exhibited the highest upper limit (100 mg/mL, mean 28.53 ± 41.65 mg/mL, n = 11). *P. aeruginosa* showed moderate values (overall mean 7.096 ± 10.804 mg/mL) with aqueous extracts surprisingly low (0.85 ± 1.36 mg/mL, n = 6), while *E. faecalis* had extreme outliers (0–58 mg/mL) (Table 1). Notably, fresh plant latex emerged as a highly potent inhibitor with an MIC of 0.03 mg/mL (30 μg/mL) against both *S. aureus* and *E. coli*, outperforming many crude organic extracts. Furthermore, isolated flavonoids, such as the bound root flavonoids, demonstrated exceptional potency, with MICs as low as 0.039 mg/mL against *S. aureus* (Figure 3).

**TABLE 1:** Minimum Inhibitory Concentration (MIC) values of different solvent extracts against ESKAPE Pathogens.

Solvent	Organisms	n	Minimum	Maximum	Mean (mg/mL)	Std. Deviation
Ethanol	*E. coli*	5	0.189	30	16.46	16.47
Methanol		13	0.5	44	17.02	32.16
Aqueous		8	3	60	14.39	25.23
Petroleum ether		5	0	2	0.40	0.89
Chloroform		5	1	6.25	1.45	2.72
​	Overall	**7.2**	**0.9378**	**28.45**	**9.944**	**15.494**

Solvent	Organisms	n	Minimum	Maximum	Mean (mg/mL)	Std. Deviation
Ethanol	*S. aureus*	5	0.216	30	14.53	19.02
Methanol		6	0.25	42	7.20	17.04
Aqueous		7	2	60	9.28	22.39
Petroleum ether		4	0.5	-	0.125	0.25
Chloroform		4	0	2	0.5	1
​	Overall	**4.5**	**0.108**	**16**	**7.515**	**10.01**

Solvent	Organisms	n	Minimum	Maximum	Mean (mg/mL)	Std. Deviation
Ethanol	*K. pneumoniae*	4	1	44	19.72	20.78
Methanol		11	0.069	100	28.53	41.65
Aqueous		6	2	150	30.18	59.46
Petroleum ether		3	0.078	2	1.039	1.35
Chloroform		4	2	6.25	1.81	2.99
​	Overall	**5.6**	**1.0294**	**60.45**	**16.2558**	**25.246**

Solvent	Organisms	n	Minimum	Maximum	Mean (mg/mL)	Std. Deviation
Ethanol	*P. aeruginosa*	2	0.125	40	20.06	28.19
Methanol		12	0.125	63	12.72	20.87
Aqueous		6	2	3	0.85	1.36
Petroleum ether		5	0	2	0.4	0.89
Chloroform		5	0	6.25	1.45	2.71
​	Overall	**6**	**0.45**	**22.85**	**7.096**	**10.804**

Solvent	Organisms	n	Minimum	Maximum	Mean (mg/mL)	Std. Deviation
Ethanol	E. faecalis	1	0	58	29	41.01
Chloroform		1	0	0	-	-
​	Overall	**1**	**0**	**29**	**29**	**41.01**

The bold values indicate the most potent inhibitory or bactericidal concentrations (lowest MIC/MBC values) recorded for each specific organism-solvent combination across the synthesized data.

#### 3.3.2 Minimum bactericidal concentration (MBC)

**FIGURE 4:**
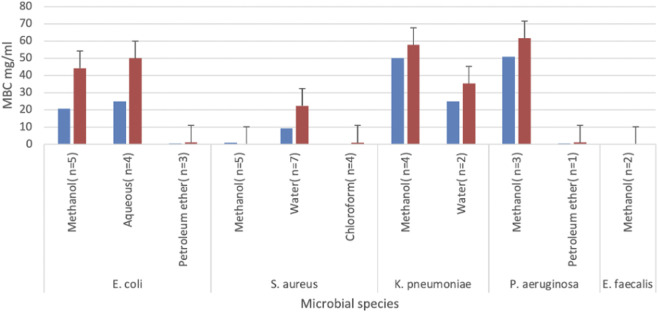
Minimum Bacterial Concentration (MBC) of plant extract solvents against ESKAPE Pathogens.

MBC values ranged from a minimum (lowest potency, highest value) of 50.81 mg/mL (*P. aeruginosa* with methanol) to a maximum potency (lowest value) of 0.1 mg/mL (*S. aureus* with methanolic extract). The highest SD was 61.59 mg/mL for *P. aeruginosa* (methanolic extract), reflecting high experimental variability (Table 2) Overall averages indicate that bactericidal concentrations are typically two to four times higher than corresponding MICs, confirming a primarily bacteriostatic nature at lower doses that transitions to bactericidal at higher concentrations. The overall averages indicate higher killing concentrations than MICs (Figure 4).

**TABLE 2:** Minimum Bactericidal Concentration (MBC) values of different solvent extracts against ESKAPE Pathogens.

Solvent	Organisms	n	Minimum	Maximum	Mean (mg/mL)	Std. Deviation
Methanol	*E. coli*	5	0	100	20.82	44.27
Aqueous		4	0	100	25	50
Petroleum ether		3	0	2	0.66	1.154
​	Overall	**4**	**0**	**67.33333333**	**15.49333333**	**31.808**

Solvent	Organisms	n	Minimum	Maximum	Mean (mg/mL)	Std. Deviation
Methanol	*S. aureus*	5	0	0.5	1.0	0.22
Water		7	0	60	9.3	22.39
Chloroform		4	0	2	0.5	1
​	Overall	**5.33**	**0**	**20.8333**	**3.6**	**7.87**

Solvent	Organisms	n	Minimum	Maximum	Mean (mg/mL)	Std. Deviation
Methanol	*K. pneumoniae*	4	0.052	100	50.01	57.72
Water		2	0	50	25	35.35
​	Overall	**3**	**0.026**	**75**	**37.505**	**46.535**
​	Overall	​	​	​	​	​

Solvent	Organisms	n	Minimum	Maximum	Mean (mg/mL)	Std. Deviation
Methanol	*P. aeruginosa*	3	0.25	125	50.81	61.59
Petroleum ether		1	0	2	0.66	1.15
​	Overall	**2**	**0.125**	**63.5**	**25.735**	**31.37**

Solvent	Organisms	n	Minimum	Maximum	Mean (mg/mL)	Std. Deviation
Methanol	E. faecalis	2	0	0.5	0.25	0.35
​	Overall	**2**	**0**	**0.5**	**0.25**	**0.35**

The bold values indicate the most potent inhibitory or bactericidal concentrations (lowest MIC/MBC values) recorded for each specific organism-solvent combination across the synthesized data.

For *E. coli*, methanolic extracts reached 100 mg/mL (mean 20.82 ± 44.27 mg/mL, n=5) while petroleum ether extracts remained low (mean 0.66 ± 1.15 mg/mL, n=3). *S. aureus* showed tight clustering around 0.5 mg/mL for chloroform (mean 0.5 ± 1.0 mg/mL), with petroleum ether extracts at 0.125 ± 0.25 mg/mL (n=5 replicates), confirming low MIC variability. *K. pneumoniae* required up to 100 mg/mL for methanolic extracts (mean 50.01 ± 57.72 mg/mL, n=4), and *P. aeruginosa* exhibited the highest variability (0.25–125 mg/mL, methanolic extracts mean 50.81 ± 61.59 mg/mL, n = 3), confirming that bactericidal endpoints are harder to achieve against resilient Gram-negative bacteria. In contrast, MBCs for Gram-positive isolates like *E. faecalis* clustered at lower levels, typically around 0.25 mg/mL (0–0.5 mg/mL, mean 0.25 ± 0.35 mg/mL, n = 2) (Table 2).

#### 3.3.3 Disk diffusion (DD)

The results from disk diffusion tests offer a comparative, semi-quantitative evaluation of the inhibitory activity of the plant extracts, and this method is most informative in assessing the potency of solvents and plant parts. From 27 studies, methanolic and ethanolic extracts generally showed larger inhibition zones, often ranging from 0 to 48 mm against *E. coli* and *S. aureus*, particularly at higher concentrations, whereas non-polar solvents such as hexane or petroleum ether showed very small or no inhibition zones, possibly because of the inability of lipophilic metabolites to diffuse in agar media.

DD zones showed a global minimum of 0 mm (multiple extracts, e.g., chloroform extracts on *P. aeruginosa*) and a maximum of 48 mm (ethanolic extracts on *S. aureus*; mean 30.67 ± 15.31 mm). Key organism ranges included *E. coli* 0–36 mm (ethanolic extracts, mean 28.31 ± 7.51 mm), *K. pneumoniae* 0–44 mm (ethanolic extracts, mean 32.97 ± 15.61 mm), *P. aeruginosa* 0–40 mm (ethanolic extracts), and *E. faecalis* 0–58 mm (ethanolic extracts); petroleum ether peaked at 17.66 mm (*E. coli*, mean 8.19 ± 8.90 mm). High variability noted in ethanolic extracts (e.g., *S. aureus* SD 15.31 mm) confirms the ethanolic extract’s superior activity (Supplementary Table S4).

*E. coli* and *S. aureus* showed high susceptibility; *P. aeruginosa* was generally more resilient, though methanolic and ethanolic preparations still achieved significant zones of 23–24 mm. Conversely, several studies reported that aqueous extracts often failed to produce inhibitory zones in the DD, resulting in frequent “non-responder” status. Notably, fresh plant latex demonstrated exceptional potency in DD assays, producing a 27.93 mm zone against *E. coli*, which outperformed standard antibiotics like ceftriaxone in the same trial.

#### 3.3.4 Well diffusion (WD)

The results in the WD assays, performed extensively for crude and fractionated preparations, generally showed patterns in accordance with the disk diffusion method, albeit with greater sensitivity to the amount of extract loaded and the volume diffused. Ethanolic, methanolic, and hydroalcoholic extracts of *E. hirta* displayed inhibition zones in the WD method against a range of pathogens, including MDR *E. coli*, ESBL-producing *K. pneumoniae*, and *A. baumannii*, with zones generally greater in diameter than those observed in the disk diffusion method, presumably because of the greater volume diffused and better interaction with the agar. On the other hand, aqueous and decoction-based preparations showed variable results, with some studies demonstrating small inhibition zones for Gram-positive cocci, whereas in other studies, no inhibition was observed, presumably because of the instability of heat-labile metabolites in aqueous solutions.

WD produced smaller zones overall (0–18 mm), with mean of methanolic extracts on *E. coli* with 11.35 ± 2.33 mm (n = 2) exceeding aqueous extracts (7.4 ± 6.89 mm, n = 5); *S. aureus* aqueous extracts averaged 4.75 ± 5.12 mm (n = 4), and *K. pneumoniae* methanolic extracts reached 13.15 ± 4.03 mm (n = 2) versus aqueous extracts 7 ± 9.96 mm (n = 5). The effect of the aqueous extracts on *P. aeruginosa* was modest (4 ± 5.66 mm, n = 5), and the mean for the methanolic extracts on *E. faecalis* averaged 5 ± 7.07 mm (n = 2), confirming higher sensitivity to extract diffusion (Supplementary Table S5). Overall, the data suggest that while DD is highly effective for rapid solvent screening, WD remains a critical tool for quantifying the activity of viscous or volume-dependent extracts like fresh juice and crude macerates.

## 4 Discussions

*E. hirta* (Euphorbiaceae), commonly known as asthma plant, dudhi, or snakeweed, is a prostrate, annual botanical drug native to tropical and subtropical America that has naturalised across South and Southeast Asia, Africa, and the Pacific Islands. It is used in Ayurveda, Siddha, Unani, and African folk medicine for respiratory ailments (asthma, bronchitis), dysentery, skin infections, and wounds. Its milky latex is particularly valued for treating open wounds, boils, and dysentery due to its purported antimicrobial and anti-inflammatory properties. *E. hirta* is known to be rich in flavonoids, such as quercetin, rutin, and epicatechin gallate/ECG; phenolic acids, like gallic, chlorogenic, and caffeic; triterpenoids, such as taraxerol and amyrins; tannins; phytosterols; and volatile oils, with aerial parts and latex being the chief reservoirs of medicinal properties. Recent studies have validated the pharmacological potential of *E. hirta*, with activities such as antibacterial effects on MDR pathogens, antioxidant activities via NO scavenging, anti-inflammatory activities, JAK/STAT and MAPK pathway modulations, α-glucosidase inhibition for antidiabetic effects, and promotion of wound healing via collagen synthesis and fibroblast proliferation. *E. hirta* is rich in flavonoids (quercetin, rutin, epicatechin gallate/ECG), phenolic acids (gallic, chlorogenic, caffeic), triterpenoids (taraxerol, amyrins), tannins, phytosterols, and volatile oils, with the aerial parts and latex serving as primary medicinal reservoirs. The broad range of activities *E. hirta* possesses has been backed up by recent research. It has displayed antimicrobial activity against multidrug-resistant strains of infection. Due to its nitric oxide-scavenging capabilities, it has antioxidant properties as well. The mechanisms behind its anti-inflammatory effects include regulation of MAPK and JAK/STAT pathways*. E. hirta* also displays anti-diabetic capabilities by inhibiting α-glucosidase. Through stimulation of collagen production and fibroblast proliferation, it may also have wound-healing properties. Because of its availability, low-cost, and high safety level, *E. hirta* is an ideal candidate for “One Health” strategies addressing antimicrobial resistance through plant-based alternatives. (Kumar et al., 2010).

The systematic analysis of the 27 *in vitro* studies shows that *E. hirta* possesses significant antimicrobial potential; its pharmacological potential are highly variable and contingent upon extraction parameters. Potency ranges from an exceptional 0.00195 mg/mL against *K. pneumoniae* to a largely inactive 100 mg/mL for certain crude methanolic extracts. Most extracts are primarily bacteriostatic at lower concentrations, only reaching bactericidal status at doses two to four times higher than the MIC (MBC/MIC ratio ≤4).

*E. hirta* has shown a wide range of antibacterial activity against several bacterial infections. MIC values as low as 0.00195 mg/mL or 1.95 μg/mL demonstrated strong inhibitory action against *K. pneumoniae*. It also demonstrated significant action against *S. typhi* with an MIC of 0.031 mg/mL. Bactericidal actions against *S. aureus* and *E. coli* were noted. A moderate inhibitory activity was seen against *P. aeruginosa* and *K. pneumoniae* with MIC values of about 0.062 mg/mL. Antibacterial activity against *A. baumannii* and *E. faecalis* was comparatively less. The solubility of bioactive metabolites and the polarity of the solvent may have a significant impact on the variation in antibacterial activity. The pharmacological potential of ethanolic extracts with medium polarity and fresh plant latex was higher; fresh latex had an MIC of 0.03 mg/mL. Conversely, aqueous and non-polar extracts often showed very little bactericidal action. The pharmacological potential of *E. hirta* should therefore be interpreted cautiously. Standardized experimental protocols and tailored solvent systems are necessary to improve reproducibility and pharmacological potential against resistant bacteria. This is especially important for non-fermenting bacteria like *P. aeruginosa*, which naturally show higher resistance (Kumar et al., 2010; Perumal et al., 2018).

*E. coli* and *K. pneumoniae* strains were found to exhibit moderate to varying susceptibility depending on the strains and extraction techniques employed in different studies. The antimicrobial activity of these strains was found to be highly dependent on solvents, in which alcoholic and hydro alcoholic extracts were found to exhibit lower values of MIC for MDR strains of *E. coli* and *K. pneumoniae* and were found to be superior to aqueous extracts in all cases (Perumal and Mahmud, 2013). Although some of these studies reported that these strains were inhibited at very low concentrations, with the minimum value of 1.95 μg/mL for *K. pneumoniae*, whereas in some cases, higher values of 100 mg/mL were observed for MIC and MBC, which may be due to the variation in strains and extraction techniques employed in different studies (Abubakar, 2009). However, in all cases, the suppression of these strains by optimized solvents may be taken as evidence for the antimicrobial activity of *E. hirta* against the causative agents of urinary tract infections and gastrointestinal infections (Mbwale et al., 2025).

In contrast, *P. aeruginosa* has been identified as having highly variable susceptibility; whereas some studies have identified it as the least responsive pathogen, others have identified it as the most susceptible organism to methanolic extracts of *E. hirta* with an MIC as low as 0.062 mg/mL (Rajeh et al., 2010). While aqueous extracts showed minimal activity, hydroalcoholic and ethanolic extracts of *E. hirta* have shown considerable inhibitory activity, including against imipenem and meropenem-resistant isolates of *P. aeruginosa* (Sudhakar et al., 2006). In addition, isolated metabolites such as ECG and caffeic acid have been shown to have effective resistance-modifying activity, showing synergy with conventional antibiotics such as cefepime in overcoming resistance mechanisms with an average FIC index of 0.24 (Perumal et al., 2018). These results are of great importance as resistance in *P. aeruginosa* is difficult to manage in the clinic and has been associated with cell wall destruction and intracellular leakage of potassium and nucleotides (Perumal et al., 2018).

*S. aureus*, including MRSA strains, was consistently identified as one of the most sensitive organisms across multiple assays (Rajeh et al., 2010). Potency was notably high with alcoholic extracts and isolated bound root flavonoids, which achieved MICs as low as 0.039 mg/mL (Awaad et al., 2017). Interestingly, fresh plant latex showed superior or equivalent potency compared to reference drugs such as vancomycin and ciprofloxacin, exhibiting a 27.93 mm zone of inhibition and an MIC of 0.03 mg/mL against *S. aureus* (Hussain et al., 2014). Like other ESKAPE pathogens, *E. hirta* derivatives are potent adjuvants. For example, flavonoids and curcumin have been reported to significantly reduce the MICs of β-lactams and aminoglycosides against resistant *S. aureus* strains (Perumal et al., 2018).

Significantly, although less emphasized, other priority pathogens, such as *E. faecalis* and *A. baumannii,* exhibited variable susceptibility to *E. hirta* extracts. In this respect, the methanolic extracts exhibited inhibitory effects on *E. faecalis* at a 0.125 mg/mL concentration. Moreover, the use of the partially purified extracts was highly effective in inhibiting *A. baumannii*. This implies the need for the optimization of solvents and the use of purified extracts such as peptides and flavonoids in the control of highly resistant ESKAPE pathogens in order to achieve the desired outcomes (Perumal and Mahmud, 2013).

Solvent-wise, the pharmacological potential of the ethanolic fraction, followed by methanolic fraction, was more pronounced, which was in accordance with the reported phytochemical distribution of *E. hirta*, including flavonoids such as quercetin, rutin, kaempferol, phenolic metabolites like gallic, caffeic acids, tannins, and triterpenoids like α/β-amyrin, which are optimally extracted with solvents of medium polarity, may be known to interfere with bacterial membranes, efflux pumps, and exhibit observed *in vitro* interactions with conventional antibiotics. Aqueous and hydro alcoholic extracts showed varying degrees of pharmacological potential, which might be due to incomplete extraction of lipophilic metabolites along with good recovery of polar glycosides, while petroleum ether and chloroform fractions were ineffective, indicating a minor contribution of lipophilic metabolites to the antibacterial activity (Kumar et al., 2010; Perumal et al., 2018).

Varying methodology from study to study, from crude screening by DD/WD to quantitative determination of MIC/MBC. The assays are associated with moderate heterogeneity, but in all cases, trends are observed: Gram-negative enteric pathogens (*E. coli, K. pneumoniae*) are generally more predictable in their response than non-fermenters (*P. aeruginosa, A. baumannii*), and susceptible reference strains are generally easier to inhibit than multidrug-resistant clinical isolates (Perumal and Mahmud, 2013; Singh and Kumar, 2013).

Other research works assessed the synergistic potential of *E. hirta* extracts in combination with conventional antibiotics such as cefepime, ciprofloxacin, erythromycin, and lincomycin. The observed synergistic interactions position *E. hirta* as a promising resistance-modifying agent capable of restoring antibiotic susceptibility, with fractional inhibitory concentration indices. In a checkerboard titration method, the isolated ECG from the aerial parts of the plant was found to show significant synergism when combined with cefepime against resistant clinical isolates of *P. aeruginosa*, where the average FIC index was 0.24, and the MIC was reduced by as much as 32-fold (Perumal et al., 2018). In addition, purified whole plant acetone extracts, when mixed with honey and ciprofloxacin, were found to possess a remarkably low FIC index value of 0.02, confirming the potentiation effect against MDR *P. mirabilis* (Oseni et al., 2021). ECG and caffeic acid were found to be synergistic when acting as antibacterial agents since the mechanism of action was related to cell wall disruption and the leakage of vital cellular contents such as potassium ions, sodium ions, proteins, and nucleotides(Perumal et al., 2018).

A primary oversight in the literature is the failure to account for Pan-Assay Interference Compounds (PAINS). *E. hirta* is rich in ‘promiscuous’ polyphenols, including quercetin, kaempferol, and tannins, that frequently produce false-positive results through non-specific membrane perturbation or protein aggregation. These artifacts are easily misconstrued as targeted mechanisms, such as efflux pump inhibition (Bolz et al., 2021). Consequently, reported antimicrobial activities of crude extracts must be interpreted with caution, as many observed effects likely reflect experimental artifacts rather than specific pharmacological targets.

**FIGURE 5:**
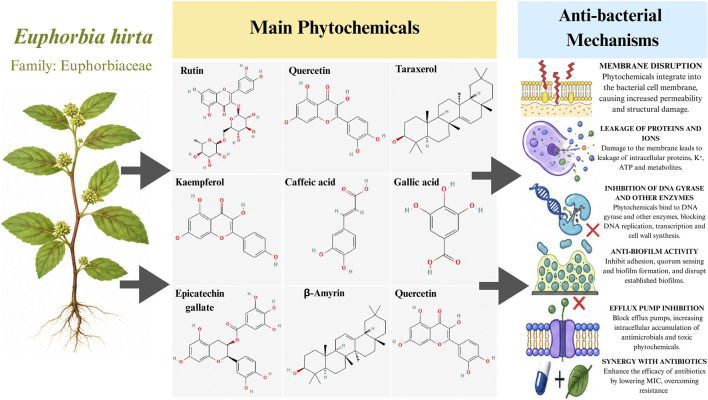
Hypothetical pharmacological effects and chemical structures of major metabolites identified in E. hirta. Chemical structures were obtained from PubChem database (https://pubchem.ncbi.nlm.nih.gov). Mechanistic illustrations and biomedical icons were adapted from Servier Medical Art (Smart Servier; https://smart.servier.com). The final figure layout and graphical arrangement were prepared by the authors using Canva (https://www.canva.com).

The antimicrobial effects of *E*. *hirta* are fundamentally driven by its ability to act as a resistance-modifying agent through several hypothesized pharmacological pathways (Perumal et al., 2018). Bioactive metabolites, such as caffeic acid and epicatechin 3-gallate (ECG), have been reported to break cell walls and compromise the integrity of the bacterial cytoplasmic membrane (Perumal et al., 2015). The irreversible leakage of essential intracellular components, such as proteins, nucleotides, and potassium and sodium ions, is confirmed by mechanistic investigations using SEM and physiological assays. Beyond causing direct membrane damage, the plants phytochemical matrix metabolites, especially flavonoids like quercetin and kaempferol and terpenoids, are reported to interfere with bacterial efflux pumps and prevent the formation of biofilms, hence neutralizing ESKAPE pathogen survival strategy (Perumal and Mahmud, 2013). Studies have demonstrated potential synergistic potentiation of conventional antibiotics by the restoration of cefepime activity against MDR *P. aeruginosa* (FICI 0.24) and ciprofloxacin against *P. mirabilis* (FICI 0.02) (Oseni et al., 2021). Hypothetical pharmacological effects and chemical structures of major metabolites identified in *E. hirta* are summarized in Figure 5. These pathways are based on *in vitro* observations of membrane perturbation and reported interactions; however, definitive molecular targets remain unproven. A critical caveat is the predominance of tannins and quercetin, which are recognized PAINS which may lead to non-specific “promiscuous” activity in laboratory assays that may not translate to specific clinical relevance.

Apart from *E. hirta*, several plant-derived substances have been found to possess appreciable antibacterial potential against ESKAPE pathogens, with some substances having higher potential than others. A comparative study showed that by fractions *of Nauclea diderrichii* (Laurent) Merr. *Exhibited greater potential than E. hirta in inhibiting certain bacterial strains, achieving a lower MIC of 7.8 μg/mL compared to 31.25 μg/mL for E. hirta* (Agbebi et al., 2022). In a similar study, *Cyclea peltata* Hook. F. & Thomson was found to be more active than *E. hirta* in inhibiting a wide range of human pathogens in a disc diffusion assay by achieving higher levels of inhibition (Raja et al., 2011). Among members of the same family, *E. hirta* showed higher potential than *Euphorbia thymifolia* L.*, E. granulate* Forssk., and *E. helioscopia* L. in inhibiting *Staphylococcus aureus* and *Escherichia coli* by achieving larger zones of inhibition with lower MICs for fresh latex and ethanolic extracts of *E. hirta* (Farooq et al., 2014). Additionally, although *E. hirta* has shown potent synergy with the antibiotic cefepime (FICI 0.24), other plants such as *Plumbago zeylanica* L. and *Terminalia arjuna* (Roxb. Ex DC.) Wight & Arn.have also shown potent inhibitory and synergistic effects with conventional antibiotics, making the *Euphorbiaceae* and other medicinal plant groups critical reservoirs for resistance-modifying antibiotics (Kaushik et al., 2025).

*E. hirta* possesses significant antibacterial potential, according to the overall analysis of organism-based and solvent-based extractions. Medium-polarity solvents (methanol/ethanol) and live plant latex exhibit significant antibacterial activity. These extraction methods successfully recover bioactive metabolites like terpenoids, phenolic acids (caffeic acid), and flavonoids (quercetin, kaempferol, EGC). Methodological variations also impact antimicrobial outcomes because extraction efficiency depends on the method employed. While 66.7% of studies use traditional techniques like maceration, advanced methods like Accelerated Solvent Extraction (ASE) enhance bioactive chemical recovery.

A significant “susceptibility gap” has been noticed among the isolates; standard ATCC reference strains are often inhibited at doses 10–100 times lower than those of clinical multidrug-resistant pathogens. This discrepancy points towards the acquired resistance mechanisms in clinical isolates, such as the mecA gene in *S. aureus* and MexXY-OprM efflux pumps in *Pseudomonas aeruginosa*. Ultimately, these findings highlight the critical need for uniform testing procedures and optimal solvent systems to bridge the gap between laboratory results and clinical reality.

Limitations and Translational challenges: Despite the promising potential of E. hirta, several critical limitations restrict its translational utility. A primary limitation is the lack of clinical evidence in terms of human clinical trials; the present evidence is limited to experimental *in vitro* and pre-clinical studies (Rajeh et al., 2012; Yuet Ping et al., 2013). Furthermore, there is insufficient data on the safety of oral and topical applications conducted on toxicity models. The brine shrimp lethality assays suggest low acute toxicity in most cases; specific plant parts have demonstrated potential toxicity at higher concentrations, highlighting the need for systematic validation in human mammalian cell lines to establish a definitive therapeutic index and lethal dose (LD_50_). Furthermore, variations in the phytochemistry content in *E. hirta* create additional problems related to the use of different extraction techniques, standardization and reproducibility (Rajeh et al., 2012; Yuet Ping et al., 2013; Tripathi et al., 2022). Only eight (29.6%) of the 27 included studies presented quantitative data backed by thorough statistical analysis. Typically, the statistical data were displayed as standard error or mean ± SD. The majority of studies used descriptive findings. Measures of reproducibility and repeatability were also not well reported. The lack of bioassay-guided fractionation technique (HPLC, GC-MS) data (Perumal et al., 2018; Oseni et al., 2021), which is essential for the isolation and validation of exact bioactive agents, guarantees repeatability and methodically assesses the antibiotics’ synergistic reactivation (Rajasudha and Manikandan, 2019; Chin et al., 2023). Additionally, most studies have failed to adhere to established CLSI or EUCAST antimicrobial susceptibility guidelines (M7, CLSI 2024), thereby lowering the strength of evidence and restricting the comparability of results across different research settings. Addressing these gaps through pharmacokinetic profiling, bioassay-guided fractionation, and the use of standardised testing protocols is essential to bridge the gap between *in vitro* findings and viable clinical applications (Cusack et al., 2019). *E. hirta* possesses metabolites that are commonly known to be part of the PAINS family and can result in assay interference. As such, the biological activity that has been reported from *in vitro* testing should not be assumed to have any pharmacological implications. Any biological activity, including the inhibition of efflux pumps, should be treated as theoretical (Baell and Nissink, 2018; Newman, 2021).

## 5 Conclusion

The increasing problem of AMR, especially in the case of ESKAPE bacteria, necessitates novel approaches to antimicrobial therapy. *E. hirta* seems like a potential candidate in this regard due to its antimicrobial and resistance-modifying effects on ESKAPE pathogens. According to 27 studies performed *in vitro,* the optimal antimicrobial effects can be observed through proper extraction techniques with medium-polarity solvents and low MIC values. In addition to its inhibitory effect on bacterial growth, *E. hirta* is capable of decreasing the effects of resistance. This may take place via biofilm breakdown and weakening of the cell wall. The weakening of the wall causes leakage of ions and nucleotides from bacteria. Therefore, the total antibacterial effect may increase. Nevertheless, the usage of *E. hirta* in clinical practice needs much work to get rid of the existing obstacles. First of all, more studies need to be done in terms of the enhancement of the extract preparation process and standardisation. Secondly, isolation of active metabolites, pharmacokinetics, and *in vivo* investigations need to be conducted. Additionally, nanotechnology and combination treatment might make botanical drug remedies more economically viable. The One Health concept will be promoted with the help of *E. hirta*.

## Data Availability

The original contributions presented in the study are included in the article/Supplementary Material, further inquiries can be directed to the corresponding authors.
