# Supplementary material for: Euphorbia hirta L. as a potential resistance-modifying agent against ESKAPE pathogens: a systematic review
Source: Front Pharmacol. 2026 Jul 9;17:1862731. doi: 10.3389/fphar.2026.1862731 (PMC13391868; doi:10.3389/fphar.2026.1862731)
Supplement: Supplementary file 1 [file Supplementaryfile1.docx]

**Supplementary material**

**Supplementary Table 1.**

**Database-wise search syntax, keywords, and Boolean operators used for systematic literature retrieval.**

| **Sl. No.** |  | **Keywords** |
| --- | --- | --- |
| **1** | **Organisms** | *E. coli, Escherichia coli, P. aeruginosa, Pseudomonas aeruginosa, K. pneumoniae, Klebsiella pneumoniae, A. baumannii, Acinetobacter baumannii*, ESKAPE. |
| **2** | ***Euphorbia hirta*** | Euphorbia [MeSH Terms], E. hirta, Euphorbia hirta, Euphorbia capitata Lam., Euphorbia pilulifera Jacq., Chamaesyce hirta, Asthma plant, dove milk, garden spurge, Semi erect euphorbia, Snake weed, Dudhi, Dugdhika, Pusitoa, Achchegida, Haalukudi, Haaluni, Kempu Nene Akki, Kempu Neneakkee Soppu, Marri Jevanig,Dudhi, Dudnali, Govardhan, Mothidudhi, Nelapalai, Alapatikam, Ammatti, Amumpatchaiyarissi, Ciivitu, Cintialatcurai, Cirrilairpalatai, Cirupalatai, Cittirapalatai, Cittirappalavi, Cittiravalaya, Cutuculatti, Karaccam, Katuturatti, Nakayacceti, Nalayam, Nallammanpaccarici, Nulaccukam, Palatai, Parcorrikosti, Parcurri, Patchaiyarissi, Pulika |
| **3** | **Antimicrobial** | Antimicrobial activity, Antimicrobial Potential, Antibacterial activity, Antibacterial potential, antimicrobial resistance, AMR, Antibiotic resistance, Multidrug Resistance, MDR, Pathogen resistance, microbial resistance, Resistance to Antibiotics, Pharmaco resistance, Bacterial Priority pathogens. |

| **Search Databases:** |
| --- |
| **PUBMED** (<https://pubmed.ncbi.nlm.nih.gov/>), |
| ("Klebsiella pneumoniae" OR "K. pneumoniae" OR "Acinetobacter baumannii" OR "A. baumannii" OR "Pseudomonas aeruginosa" OR "P. aeruginosa" OR "Escherichia coli" OR "E. coli" OR ESKAPE) AND ((Euphorbia [MeSH Terms]) OR "E. hirta" OR "Euphorbia hirta" OR "Asthma plant" OR "garden spurge" OR "Snake weed" OR Dudhi OR Dugdhika OR Pusitoa OR Achchegida OR Haalukudi OR Haaluni OR "Kempu Nene Akki" OR "Kempu Neneakkee Soppu" OR "Marri Jevanige" OR Dudhi OR Dudnali OR Govardhan OR Mothidudhi OR Nelapalai OR Alapatikam OR Ammatti OR Amumpatchaiyarissi OR Ciivitu OR Cintialatcurai OR Cirrilairpalatai OR Cirupalatai OR Cittirapalatai OR Cittirappalavi OR Cittiravalaya OR Cutuculatti OR Karaccam OR Katuturatti OR Nakayacceti OR Nalayam OR Nallammanpaccarici OR Nulaccukam OR Palatai OR Parcorrikosti OR Parcurri OR Patchaiyarissi OR Pulika OR Feiyangcao OR Labeinah OR "Ambin jantan" OR "Daun biji kacang" OR "Moluccas" OR "Nanangkaan" OR Sip OR Gunantuna OR Botobotonis OR "Mouk may" OR "Nam nom raatchasee" OR Corsuwxra OR " Euphorbea fleusentete" OR " Boro Keruie" OR "Gelang Susu" OR "Demba sindji" OR "Asthma herb" OR Tuagbono) AND (antimicrobial activity OR antimicrobial potential OR Drug resistance OR Antibiotic resistance OR Microbial resistance OR Pathogen resistance OR Multidrug resistance OR MDR OR "Resistance to antibiotics" OR Pharmacoresistance) – **78 hits** |
|  |
| **SCOPUS** (<https://www.scopus.com/>) |
| (TITLE-ABS-KEY("Klebsiella pneumoniae" OR "K. pneumoniae" OR "Acinetobacter baumannii" OR "A. baumannii" OR "Pseudomonas aeruginosa" OR "P. aeruginosa" OR "Escherichia coli" OR "E. coli" OR ESKAPE)) AND (TITLE-ABS-KEY(“E. hirta" OR "Euphorbia hirta" OR "Asthma plant" OR "garden spurge" OR "Snake weed" OR Dudhi OR Dugdhika OR Pusitoa OR Achchegida OR Haalukudi OR Haaluni OR "Kempu Nene Akki" OR "Kempu Neneakkee Soppu" OR "Marri Jevanige" OR Dudhi OR Dudnali OR Govardhan OR Mothidudhi OR Nelapalai OR Alapatikam OR Ammatti OR Amumpatchaiyarissi OR Ciivitu OR Cintialatcurai OR Cirrilairpalatai OR Cirupalatai OR Cittirapalatai OR Cittirappalavi OR Cittiravalaya OR Cutuculatti OR Karaccam OR Katuturatti OR Nakayacceti OR Nalayam OR Nallammanpaccarici OR Nulaccukam OR Palatai OR Parcorrikosti OR Parcurri OR Patchaiyarissi OR Pulika OR Feiyangcao OR Labeinah OR "Ambin jantan" OR "Daun biji kacang" OR "Moluccas" OR "Nanangkaan" OR Sip OR Gunantuna OR Botobotonis OR "Mouk may" OR "Nam nom raatchasee" OR Corsuwxra OR " Euphorbea fleusentete" OR " Boro Keruie" OR "Gelang Susu" OR "Demba sindji" OR "Asthma herb" OR Tuagbono)) AND (TITLE-ABS-KEY(antimicrobial activity OR antimicrobial potential OR Drug resistance OR Antibiotic resistance OR Microbial resistance OR Pathogen resistance OR Multidrug resistance OR MDR OR "Resistance to antibiotics" OR Pharmacoresistance)) – **8 hits** |
|  |
| **SCIENCE DIRECT** (<https://www.sciencedirect.com/>) |
| (“Klebsiella pneumoniae” OR “Acinetobacter baumannii” OR “Pseudomonas aeruginosa” OR “Escherichia coli” OR “ESKAPE”) AND (“Euphorbia hirta”) AND (“antimicrobial activity” OR “antibiotic resistance” OR” Multidrug resistance”) **-** 201 hits |
|  |
| **GOOGLE SCHOLAR** (<https://scholar.google.com/>), |
| ("Klebsiella pneumoniae" OR "K. pneumoniae" OR "Acinetobacter baumannii" OR "A. baumannii" OR "Pseudomonas aeruginosa" OR "P. aeruginosa" OR "Escherichia coli" OR "E. coli" OR ESKAPE) AND ("E. hirta" OR "Euphorbia hirta" OR "Asthma plant" OR "garden spurge" OR "Snake weed" OR Dudhi OR Dugdhika OR Pusitoa OR Achchegida OR Haalukudi OR Haaluni OR "Kempu Nene Akki" OR "Kempu Neneakkee Soppu" OR "Marri Jevanige" OR Dudhi OR Dudnali OR Govardhan OR Mothidudhi OR Nelapalai OR Alapatikam OR Ammatti OR Amumpatchaiyarissi OR Ciivitu OR Cintialatcurai OR Cirrilairpalatai OR Cirupalatai OR Cittirapalatai OR Cittirappalavi OR Cittiravalaya OR Cutuculatti OR Karaccam OR Katuturatti OR Nakayacceti OR Nalayam OR Nallammanpaccarici OR Nulaccukam OR Palatai OR Parcorrikosti OR Parcurri OR Patchaiyarissi OR Pulika OR Feiyangcao OR Labeinah OR "Ambin jantan" OR "Daun biji kacang" OR "Moluccas" OR "Nanangkaan" OR Sip OR Gunantuna OR Botobotonis OR "Mouk may" OR "Nam nom raatchasee" OR Corsuwxra OR " Euphorbea fleusentete" OR " Boro Keruie" OR "Gelang Susu" OR "Demba sindji" OR "Asthma herb" OR Tuagbono) AND (antimicrobial activity OR antimicrobial potential OR Drug resistance OR Antibiotic resistance OR Microbial resistance OR Pathogen resistance OR Multidrug resistance OR MDR OR "Resistance to antibiotics" OR Pharmacoresistance) **-** 62,400 hits |

**Supplementary Table 2**

| **Sr.no** | **Study ID** | **Plant part used** | **Collection place** | **Extraction method** | **Solvent used** | **Organism used** | **Methods** | **No. of replicates** | **Standard Antibiotic used** | **Outcome of the study** |
| --- | --- | --- | --- | --- | --- | --- | --- | --- | --- | --- |
| 1 | Singh et al., (2013) | Root, stem, leaf, and fruits | Jaipur, Rajasthan, India | Maceration - Soxhlet | Methanol, Pet. ether, Ethyl acetate | *E. coli,*  *P. aeruginosa,*  *S. aureus* | Disc diffusion, Micro broth dilution (MIC/MBC) | Triplicate (Disc); Duplicate (MIC) | Streptomycin | Methanol extract from stems showed antibacterial activity against *E. coli* with a disk diffusion zone of 17.66 mm. Lowest MIC (0.039 mg/ml) against *S. aureus*. |
| 2 | Agbebi et al., (2022) | Leaves | Ibadan, Nigeria | Maceration - Lyophilization | Methanol: Dichloromethane | *E. coli,*  *K. pneumoniae,*  *P. aeruginosa*,  *A. baumannii*,  *S. aureus* | MIC/MBC | - | Ciprofloxacin | Methanolic extract demonstrated antibacterial activity against *E. coli* and other ESKAPE pathogens. MIC and MBC values were determined using the microdilution method. |
| 3 | Mbwale et al., (2025) | Leaves, shoots, and flowers | Arusha, Tanzania | Maceration (Boiling; cold water soaking) | Water (hot/cold), Methanol | *E. coli,*  *K. pneumoniae* | Disc diffusion, | Quadruple | Gentamycin | Methanol extract is superior (12.5 mm zone) for *E. coli* ; cold water is effective against *E. coli* |
| 4 | Awaad et al., (2017) | Aerial part | Riyadh, Saudi Arabia | Percolation | 95% Ethanol | *E. coli,*  *K. pneumoniae,*  *S. aureu*s | MIC,  Well Diffusion | - | Gentamycin | *E.hirta* extract (In 95% Ethanol) exhibited antibacterial activity with MIC values of 0.0156 mg/mL for *E. coli*, 0.038 mg/mL for *K. pneumoniae*, and 0.0195 mg/mL for *S. aureu*s. Well diffusion zones were 21.4 mm, 24.4 mm, and 23.8 mm, respectively. |
| 5 | Perumal et al., (2012) | Aerial part | Penang, Malaysia | Maceration - Lyophilization | Hexane | *E. coli*,  *K. pneumoniae*,  *P. aeruginosa*,  *S. aureus*,  *S. typhi* | MIC | - | Amoxycillin  Gentamycin  Chloramphenicol  Vancomycin | MIC values ranged from 0.125 to 1 mg/mL against *E. coli*, *K. pneumoniae*, *P. aeruginosa*, and *S. aureus*. Strongest antimicrobial activity against *Salmonella typhi* with a MIC value of 0.031mg/ml. |
| 6 | Gupta et al., (2016) | Leaves | Ambala, Haryana, India | Maceration - Lyophilization | Petroleum ether | *E. coli,*  *S. aureus* | Well Diffusion | - | Cefotaxime ,  Ceftazidime,  Imipenem,  Amikacin  other antibiotics | Petroleum ether and ethanol extracts from *E. hirta* leaves demonstrated antibacterial activity with well diffusion zones of 9 mm and 6 mm against *E. coli*, and 13 mm and 3 mm against *S. aureus*. |
| 7 | Ragasa et al., (2013) | Stems,  Leaves and Roots | Sinait, Ilocos, Philippines | Chromatography | Dichloromethane | *E. coli,*  *P. aeruginosa*,  *S. aureus* | Disk Diffusion | - | Chloramphenicol | Dichloromethane extract from stems showed disk diffusion zones of 12 mm, 13 mm, and 15 mm against *E. coli, P. aeruginosa*, and *S. aureus,* respectively. |
| 8 | Singh et al., (2013) | Stems  leaves, root,  fruit | Jaipur, Rajasthan, India | Soxhlet | Methanol | *K. pneumonia* | MIC/MBC/ Disk Diffusion | - | Streptomycin | Petroleum ether, ethyl ether, ethyl acetate, and methanol extracts from various plant parts exhibited MIC values ranging from 0.039 to 0.078 mg/mL and MBC values of 0.039 to 0.078 mg/mL against *K. pneumonia.* Disk diffusion zones ranged from 12.75 to 16 mm. |
| 9 | Narayanan et al., (2011) | Leaves, stems, roots | Namakkal, Tamil Nadu, India | Soxhlet | Chloroform | *E. coli,*  *K. pneumoniae,*  *P. aeruginosa,*  *S. aureus*,  *E. faecalis* | MIC/  Disk Diffusion | - | Amoxicillin,  Amikacin,  Clindamycin,  Methicillin  and others | Chloroform, acetone, methanol, ethanol, and saponified liquid extracts showed MIC values ranging from 0.00625 to 0.058 mg/mL against *E. coli, K. pneumoniae, P. aeruginosa, S. aureus*, and *E. faecalis*. Disk diffusion zones ranged from 10 to 23 mm. |
| 10 | Permual et al., (2018) | Aerial parts | Penang, Malaysia | - | Methanol | *P. aeruginosa* | MIC | - | Cefepime | Synergy (FICI 0.24) with  Cefepime against resistant  isolates. The study evaluated antimicrobial activity using the microdilution method. |
| 11 | Raja et al., (2011) | Whole plant | Tirunelveli, Tamil Nadu, India | - | Hexane  Methanol | *E. coli,*  *S. aureus* | Disk Diffusion | - | - | Hexane and methanol extracts showed no antimicrobial zones against *E. coli* and *S. aureus*. |
| 12 | Sudharkar et al., (2006) | Aerial parts | AP, India | - | Ethanol | *E. coli,*  *P. aeruginosa,*  S*. aureus* | MIC/ Well Diffusion | - | Ampillicin | Ethanol extract exhibited MIC values of 0.189 mg/mL against *E. coli,* 0.166 against *P. aeruginosa* and 0.216 against S*. aureus.* Well diffusion zones were 21 mm and 19 mm, respectively. |
| 13 | Permual et al., (2018) | Whole plant | Penang, Malaysia | Fractionation | Methanol | *P. aeruginosa* | MIC/MBC | - | Cefepime | The methanolic extract showed MIC and MBC values of 0.063 mg/mL and 0.0125 mg/mL, respectively, against *P. aeruginosa*. The study evaluated antimicrobial potential through the microdilution method. |
| 14 | Perumal et al., (2017) | Whole plant | Penang, Malaysia | - | Methanol | *P. aeruginosa* | MIC/MBC | - | - | Methanol extract from the whole plant demonstrated MIC and MBC values of 0.063 mg/mL and 0.0125 mg/mL against *P. aeruginosa*, and 0.016 mg/mL and 0.031 mg/mL against resistant *P. aeruginosa* strains. The study has not reported mean and SD values. |
| 15 | Perumal et al., (2013) | Aerial part | - | Maceration - Lyophilization | Methanol | *E. coli,*  *P. aeruginosa*,  *K. pneumoniae,*  *S. aureus,*  *E. faecalis* | MIC/MBC | - | Cefepime | Methanol extract from aerial parts showed MIC values of 0.5 mg/mL, 0.062, 1 mg/mL, 0.125 and 0.125 mg/mL against *E. coli*, *P. aeruginosa*, *K. pneumoniae, S. aureus* and *E. faecalis* respectively. MBC values ranged from 0.25 to 1 mg/ml. |
| 16 | Upadhay et al., (2014) | Leaves | Tezpur, Assam, India | Percolation | Methanol | *E. coli,*  *K. pneumoniae,*  *P. aeruginosa,*  *S. aureus* | MIC | - | Chloramphenicol | Petroleum ether, chloroform, methanol, and water extracts exhibited MIC values ranging from 0.25 to >2 mg/mL against *E. coli, K. pneumoniae, P. aeruginosa,* and *S. aureus*. |
| 17 | Rajeh et al., (2010) | Leaves, Flowers, Stems,  and Roots | Penang, Malaysia | Maceration - Rotary Eva | Methanol | *E. coli*,  *K. pneumoniae,*  *S. aureus* | MIC/MBC/Disk Diffusion | - | Chloramphenicol | Methanolic extract from stems, leaves, roots, and flowers showed MIC/MBC values of 3.13 mg/mL for *E. coli*, 100 mg/mL for *K. pneumoniae*, and 12.5 mg/mL for *S. aureus*. Disk diffusion zones ranged from 15 to 28 mm. |
| 18 | Hussain et al., (2014) | Whole plant | Multan, Pakistan | Maceration - Lyophilization | Dichloromethane | *E. coli,*  *K. pneumoniae,*  *S. aureus* | MIC/ Disk Diffusion | - | Vancomycin,  Flucloxacillin,  Ceftriaxone,  Levofloxacin  And others | Dichloromethane, ethanol, methanol, and water extracts demonstrated MIC values of 0.03 mg/mL, 0.15 mg/mL, and 0.060 mg/mL with disk diffusion zones of 27.93 mm, 21.93 mm, and 25.38 mm against *E. coli, K. pneumoniae,* and *S. aureus*. |
| 19 | Oseni et al., (2021) | Whole plant | Ondo state, Nigeria, Africa | Chromatography | Acetone | *E. coli*  *K. pneumoniae,*  *P. aeruginosa,*  *S. aureus* | MIC/MBC | - | Ciprofloxacin,  Gentamycin,  Streptomycin,  Amoxicillin and others | Acetone extract exhibited MIC values of 50 mg/mL, 25 mg/mL, 50 mg/mL, and 6.25 mg/mL against *E. coli, K. pneumoniae, P. aeruginosa*, and *S. aureus*. MBC values ranged from 25 to 100 mg/mL. |
| 20 | Alhlale et al., (2019) | Stem and leaves | Ibb city, Yemen | Rotary Evaporation | Methanol  and Aqueous | *E. coli,*  *P. aeruginosa,*  *S. aureus* | Well Diffusion | - | - | Methanolic extract demonstrated well diffusion zones of 6-19.7 mm for *E. coli*, 6.2-10.9 mm for *P. aeruginosa*, and 7.1-13.7 mm for *S. aureus*. Aqueous extract demonstrated well diffusion zones of 6.1-17.7 mm for *E. coli*, 6-13.1 mm for *P. aeruginosa*, and 6.1-13.3 mm for *S. aureus*. |
| 21 | Abubakar, (2009) | Whole plant | Yola, Adamawa, Africa | Rotary Evaporation | Hexane  Methanol  Water | *E. coli,*  *K. pneumoniae* | MIC/MBC/ Well Diffusion | - | Amoxicillin | Aqueous extract gave a superior yield and activity. Aqueous extracts showed MIC at 25 - 50 mg/ml and MBC from 50-100 mg /ml. Well diffusion was 18 mm. |
| 22 | Tran et al., (2020) | Whole plant | Dong Nai Province, Vietnam | Maceration - rotary evaporator | Methanol | *E. coli,*  *P. aeruginosa*,  *S. aureus* | Disk Diffusion | - | - | Methanol, petroleum ether, chloroform, ethyl acetate, and butanol extracts exhibited disk diffusion, showing no zones against *E. coli, P. aeruginosa*, and *S. aureus*. |
| 23 | Patel et al., (2014) | Whole plant | Taranga, Danta, Ambaji, Jassore, Vireshwar, Vijaynagar, India | Rotary Evaporation | Petroleum ether, acetone, and methanol | *E. coli;*  *K. pneumoniae,*  *P. aeruginosa* | MIC/  Disk Diffusion | - | - | Antibacterial activity (MIC 12.5 µg/µL) against *K. pneumoniae*, (25 µg/µL) against *E. coli*, *P. aeruginosa*. The disk diffusion ranged from 8 to 16 mm. |
| 24 | K Suresh et al., (2008) | Leaves | Namakkal, Erode, Tamil Nadu, India | Rotary Evaporation | Chloroform  Water | *E. coli,*  *K. pneumoniae,*  *P. aeruginosa,*  *S. aureus* | Well Diffusion | - | - | Chloroform and water extracts demonstrated well diffusion zones of 2.4-3.4 mm for *E. coli*, 2.6-3 mm for *K. pneumoniae*, 2.8-4 mm for *P. aeruginosa*, and 2.6-4 mm for *S. aureus*. The study has mentioned phytochemical analysis. |
| 25 | Tona et al., (1999) | Whole plant | Kinshasa, Congo | Maceration-Decoction | Water | *E. coli,*  *K. pneumoniae,*  *P. aeruginosa,*  *S. aureus* | MIC | - | Chloramphenicol | Aqueous extract from maceration-decoction showed MIC values of 3+ for *E. coli* and *P. aeruginosa*, and 4+ for *K. pneumoniae* and *S. aureus.* |
| 26 | Srinivasan et al., (2000) | Whole plant | Maruthamalai Hills of Western Ghats, Tamil Nadu | - | Water | *E. coli*  *K. pneumoniae,*  *P. aeruginosa,*  *S. aureus* | Well Diffusion | - | - | Aqueous extract demonstrated antibacterial activity against E*. coli, K. pneumoniae, P. aeruginosa*, and *S. aureus* using the well diffusion method. Specific inhibition zone measurements were not reported. |
| 27 | Ndossi et al., (2016) | Whole plant | Nambala and Akeri, Tanzania | Rotary evaporator | Methanol,  ethyl acetate and chloroform | *E. coli,*  *K. pneumoniae,*  *P. aeruginosa* | MIC | - | Gentamycin | Methanolic, ethyl acetate, and chloroform extracts of *E. hirta* showed MIC values ranging from 1.56 to 6.25 mg/mL against *E. coli,* 1.56 to 6.25 mg/mL *K. pneumoniae*, and 3.12 to 6.25 against *P. aeruginosa*. |

**Supplementary table 2: Characteristics of included studies. This table summarises the key methodological details of the studies included in the systematic review, highlighting the diversity in plant material processing and target pathogen.**

**Supplementary Table 3**

| **ToxR Tool - Toxicological data reliability assessment tool** | | | | | | | | | | | | | | | | | | | | | |
| --- | --- | --- | --- | --- | --- | --- | --- | --- | --- | --- | --- | --- | --- | --- | --- | --- | --- | --- | --- | --- | --- |
| **Reliability assessment of in vitro toxicity studies** | | | | | | | | | | | | | | | | | | | | | |
| **Authors:** | Kamran Zaman†*, Shivani Tendulkar†, Kalesh Karun, Flemin Felix, Kranthi Kiran Akula, Nidhi Hiremath, Surthi Ravedar, Tejaswini Salunkhe, Jainabbi Patel, Asif Kavathekar, Jyothi Bhat* | | | | | | | | | | | | | | | | | | | | |
| **Title:** | *Euphorbia hirta* Linn. as a Potential Resistance-Modifying Agent Against ESKAPE Pathogens: A Systematic Review | | | | | | | | | | | | | | | | | | | | |
|  | **Criteria Group I: Test substance identification** | | | | **Criteria Group II: Test system characterisation** | | | **Criteria Group III: Study design description** | | | | | | **Criteria Group IV: Study results documentation** | | | **Criteria Group V: Plausibility of study design and data** | | | **Score** | **Sub score** |
| Study ID | Was the test substance identified? | Is the purity of the substance given? | Is information on the source/origin of the substance given? | Is all information on the nature and/or physico-chemical properties of the test item given, which you deem indispensable for judging the data (see explanation for examples)? | Is the test system described? | Is information given on the source/origin of the test system? | Are necessary information on test system properties, and on conditions of cultivation and maintenance given? | Is the method of administration given (see explanations for details)? | Are doses administered or concentrations in application media given? | Are frequency and duration of exposure as well as time-points of observations explained? | Were negative controls included (give also point, if not necessary, see explanations)? | Were positive controls included (give also point, if not necessary, see explanations)? | Is the number of replicates (or complete repetitions of experiment) given? | Are the study endpoint(s) and their method(s) of determination clearly described? | Is the description of the study results for all endpoints investigated transparent and complete? | Are the statistical methods for data analysis given and applied in a transparent manner (give also point, if not necessary/applicable, see explanations)? | | Is the study design chosen appropriate for obtaining the substance-specific data aimed at (see explanations for details)? | Are the quantitative study results reliable (see explanations for arguments)? | **0-1** | **Total** |
| Study_1 | 1 | 0 | 1 | 1 | 1 | 1 | 1 | 1 | 1 | 1 | 1 | 1 | 1 | 1 | 1 | 0 | | 1 | 1 | 16 | 16/18 |
| Study_2 | 1 | 0 | 1 | 1 | 1 | 1 | 1 | 1 | 1 | 1 | 1 | 1 | 0 | 1 | 1 | 0 | | 1 | 1 | 15 | 15/18 |
| Study_3 | 1 | 0 | 1 | 1 | 1 | 0 | 0 | 1 | 1 | 0 | 1 | 1 | 1 | 1 | 1 | 1 | | 1 | 1 | 14 | 15/18 |
| Study_4 | 1 | 0 | 1 | 1 | 1 | 1 | 1 | 1 | 1 | 1 | 1 | 1 | 0 | 1 | 1 | 0 | | 0 | 1 | 16 | 16/18 |
| Study_5 | 1 | 0 | 1 | 1 | 1 | 1 | 1 | 1 | 1 | 1 | 1 | 1 | 1 | 1 | 1 | 1 | | 1 | 1 | 17 | 17/18 |
| Study_6 | 1 | 0 | 1 | 1 | 1 | 1 | 1 | 1 | 1 | 1 | 1 | 1 | 1 | 1 | 1 | 1 | | 1 | 1 | 17 | 17/18 |
| Study_7 | 1 | 0 | 1 | 1 | 1 | 1 | 1 | 1 | 1 | 1 | 1 | 1 | 1 | 1 | 1 | 1 | | 1 | 1 | 17 | 17/18 |
| Study_8 | 1 | 0 | 1 | 1 | 1 | 1 | 1 | 1 | 1 | 1 | 1 | 1 | 1 | 1 | 1 | 1 | | 1 | 1 | 17 | 17/18 |
| Study_9 | 1 | 0 | 1 | 1 | 1 | 1 | 1 | 1 | 1 | 1 | 1 | 1 | 1 | 1 | 1 | 1 | | 1 | 1 | 17 | 17/18 |
| Study_10 | 1 | 0 | 1 | 1 | 1 | 0 | 1 | 1 | 1 | 1 | 1 | 1 | 1 | 1 | 1 | 0 | | 1 | 1 | 15 | 15/18 |
| Study_11 | 1 | 0 | 1 | 0 | 1 | 1 | 0 | 1 | 1 | 1 | 0 | 0 | 0 | 1 | 1 | 0 | | 1 | 0 | 10 | 10/18 |
| Study_12 | 1 | 0 | 1 | 0 | 1 | 1 | 0 | 1 | 1 | 0 | 0 | 1 | 1 | 1 | 1 | 0 | | 1 | 1 | 12 | 12/18 |
| Study_13 | 1 | 0 | 1 | 0 | 1 | 1 | 1 | 1 | 1 | 1 | 1 | 1 | 1 | 1 | 1 | 0 | | 1 | 1 | 15 | 15/18 |
| Study_14 | 1 | 0 | 1 | 0 | 1 | 1 | 1 | 1 | 1 | 1 | 1 | 1 | 1 | 1 | 1 | 1 | | 1 | 1 | 16 | 16/18 |
| Study_15 | 1 | 0 | 1 | 1 | 1 | 1 | 1 | 1 | 1 | 1 | 1 | 1 | 1 | 1 | 1 | 1 | | 1 | 1 | 17 | 17/18 |
| Study_16 | 1 | 0 | 1 | 0 | 1 | 1 | 1 | 1 | 1 | 1 | 1 | 1 | 1 | 1 | 1 | 1 | | 1 | 1 | 16 | 16/18 |
| Study_17 | 1 | 0 | 1 | 0 | 1 | 1 | 1 | 1 | 1 | 1 | 1 | 1 | 1 | 1 | 1 | 0 | | 1 | 1 | 16 | 16/18 |
| Study_18 | 1 | 0 | 1 | 1 | 1 | 1 | 1 | 1 | 1 | 1 | 1 | 1 | 1 | 1 | 1 | 1 | | 1 | 1 | 17 | 17/18 |
| Study_19 | 1 | 0 | 1 | 0 | 1 | 1 | 1 | 1 | 1 | 1 | 1 | 1 | 1 | 1 | 1 | 1 | | 1 | 1 | 16 | 16/18 |
| Study_20 | 1 | 0 | 1 | 0 | 1 | 1 | 1 | 1 | 1 | 1 | 1 | 1 | 1 | 1 | 1 | 1 | | 1 | 0 | 15 | 15/18 |
| Study_21 | 1 | 1 | 0 | 0 | 1 | 1 | 1 | 1 | 1 | 1 | 0 | 0 | 0 | 1 | 1 | 0 | | 1 | 0 | 11 | 11/18 |
| Study_22 | 1 | 0 | 1 | 1 | 1 | 1 | 1 | 1 | 1 | 1 | 1 | 1 | 1 | 1 | 1 | 1 | | 1 | 1 | 17 | 17/18 |
| Study_23 | 1 | 1 | 0 | 0 | 0 | 1 | 1 | 1 | 1 | 1 | 0 | 1 | 1 | 1 | 1 | 1 | | 1 | 1 | 14 | 14/18 |
| Study_24 | 1 | 0 | 1 | 0 | 0 | 1 | 1 | 1 | 1 | 1 | 0 | 0 | 1 | 1 | 1 | 0 | | 1 | 0 | 11 | 11/18 |
| Study_25 | 1 | 0 | 1 | 0 | 1 | 1 | 1 | 1 | 1 | 0 | 0 | 0 | 0 | 1 | 1 | 0 | | 1 | 0 | 10 | 10/18 |
| Study_26 | 1 | 0 | 1 | 0 | 1 | 1 | 1 | 1 | 1 | 1 | 0 | 0 | 0 | 1 | 1 | 0 | | 1 | 0 | 11 | 11/18 |
| Study_27 | 1 | 0 | 1 | 0 | 1 | 1 | 1 | 1 | 1 | 1 | 0 | 0 | 0 | 1 | 1 | 0 | | 1 | 0 | 11 | 11/18 |

**Supplementary Table 3:** The risk of bias was evaluated using the ToxR tool. Each study was assessed across five domains and given a score of 1 for adequate or 0 for inadequate performance, with a maximum possible score of 18.

**Supplementary Table 4**

**Disk diffusion**

| **Solvent** | **Organisms** | **n** | **Minimum** | **Maximum** | **Mean (mg/ml)** | **Std. Deviation** |
| --- | --- | --- | --- | --- | --- | --- |
| **Ethanol** | *E. coli* | 3 | 21 | 36 | \| 28.31 \| \| --- \| \|  \| | 7.507 |
| **Methanol** |  | 8 | 0 | 27.93 | 11.14 | 9.6751 |
| **Aqueous** |  | 1 | 0 | 27 | 27 | - |
| **Petroleum ether** |  | 3 | 0 | 17.66 | 8.186 | 8.900 |
| **Chloroform** |  | 1 | 0 | 12.2 | 12.2 | - |
|  | **Overall** | **3.2** | **4.2** | **24.158** | **17.367** | **8.694** |
| **Solvent** | **Organisms** | **n** | **Minimum** | **Maximum** | **Mean (mg/ml)** | **Std. Deviation** |
| **Ethanol** | *S. aureus* | 1 | 0 | 48 | 30.666 | 15.307 |
| **Methanol** |  | 6 | 0 | 14 | 4.118 | 6.464 |
| **Aqueous** |  | 1 | 0 | 25.38 | 25.38 | - |
| **Petroleum ether** |  | 2 | 10.49 | 11.4 | 10.945 | 0.434 |
| **Chloroform** |  | 2 | 0 | 11.4 | 6.1 | 8.626 |
|  | **Overall** | **2.4** | **2.098** | **22.036** | **15.4418** | **7.70775** |
| **Solvent** | **Organisms** | **n** | **Minimum** | **Maximum** | **Mean (mg/ml)** | **Std. Deviation** |
| **Ethanol** | *K. pneumoniae* | 2 | 21.93 | 44 | 32.965 | 15.605 |
| **Methanol** |  | 6 | 0 | 27 | 11.985 | 9.272 |
| **Aqueous** |  | 1 | 0 | 25.38 | 25.38 | - |
|  | **Overall** | **3** | **7.31** | **32.126** | **23.443** | **12.438** |
| **Solvent** | **Organisms** | **n** | **Minimum** | **Maximum** | **Mean (mg/ml)** | **Std. Deviation** |
| **Ethanol** | *P. aeruginosa* | 1 | 0 | 40 | 40 | - |
| **Methanol** |  | 4 | 0 | 17.66 | 8.165 | 9.490 |
| **Petroleum ether** |  | 2 | 0 | 10.71 | 3.71 | 6.183 |
| **Chloroform** |  | 2 | 0 | 0 | 0 | 0 |
|  | **Overall** | **3** | **0** | **8.83** | **4.0825** | **4.745** |
| **Solvent** | **Organisms** | **n** | **Minimum** | **Maximum** | **Mean (mg/ml)** | **Std. Deviation** |
| **Ethanol** | *E. faecalis* | 1 | 0 | 58 | 0 | - |
| **Methanol** |  | 2 | 0 | 10 | 5 | 7.071 |
| **Chloroform** |  | 1 | 0 | 0 | 0 | - |
|  | **Overall** | **1.333** | **0** | **22.66** | **1.666** | **7.071** |

**Supplementary Table 4: Disk diffusion values of different solvent extracts against ESKAPE Pathogens**

**Supplementary Table 5**

**Well diffusion**

| **Solvent** | **Organisms** | **n** | **Minimum** | **Maximum** | **Mean (mg/ml)** | **Std. Deviation** |
| --- | --- | --- | --- | --- | --- | --- |
| **Ethanol** | *E. coli* | 1 | 0 | 9 | 9 | - |
| **Methanol** |  | 2 | 9.7 | 13 | 11.35 | 2.333 |
| **Aqueous** |  | 5 | 0 | 18 | 7.4 | 6.890 |
| **Petroleum ether** |  | 1 | 0 | 0 | 0 | - |
| **Chloroform** |  | 1 | 0 | 2.4 | 2.4 | - |
|  | **Overall** | **2** | **1.94** | **8.48** | **6.03** | **4.6115** |
| **Solvent** | **Organisms** | **n** | **Minimum** | **Maximum** | **Mean (mg/ml)** | **Std. Deviation** |
| **Ethanol** | *S. aureus* | 1 | 0 | 38 | - | - |
| **Aqueous** |  | 4 | 0 | 12 | 4.75 | 5.123 |
| **Petroleum ether** |  | 1 | 0 | 0 | - | - |
|  | **Overall** | **2** | **0** | **16.666** | **4.75** | **5.123** |
| **Solvent** | **Organisms** | **n** | **Minimum** | **Maximum** | **Mean (mg/ml)** | **Std. Deviation** |
| **Ethanol** | *K. pneumoniae* | 1 | 0 | 9 | 9 | - |
| **Methanol** |  | 2 | 9.7 | 13 | 13.15 | 4.030 |
| **Aqueous** |  | 5 | 0 | 18 | 7 | 9.9643 |
| **Petroleum ether** |  | 1 | 0 | 0 | 0 | - |
| **Chloroform** |  | 1 | 0 | 2.4 | 2.4 | - |
|  | **Overall** | **2** | **1.94** | **8.48** | **6.31** | **6.997** |
| **Solvent** | **Organisms** | **n** | **Minimum** | **Maximum** | **Mean (mg/ml)** | **Std. Deviation** |
| **Ethanol** | *P. aeruginosa* | 1 | 0 | 4 | 4 | - |
| **Aqueous** |  | 5 | 0 | 12 | 4 | 5.656 |
| **Petroleum ether** |  | 1 | 0 | 0 | 0 | - |
| **Chloroform** |  | 1 | 0 | 0 | 0 | - |
|  | **Overall** | **2** | **0** | **4** | **2** | **5.656** |
| **Solvent** | **Organisms** | **n** | **Minimum** | **Maximum** | **Mean (mg/ml)** | **Std. Deviation** |
| **Methanol** | *E. faecalis* | 2 | 0 | 0 | 5 | 7.071 |
| **Chloroform** |  | 1 | 0 | 0 | 0 | - |
|  | **Overall** | **1.5** | **0** | **0** | **2.5** | **7.071** |

**Supplementary Table 5: Well diffusion values of different solvent extracts against ESKAPE Pathoge**
